# Flipped classroom improves student learning outcome in Chinese pharmacy education: A systematic review and meta-analysis

**DOI:** 10.3389/fphar.2022.936899

**Published:** 2022-08-30

**Authors:** Wei Peng, Ying Xiong, Jingwen Wei, Xiuping Chen, Wenying Huai, Sike He, Dan Liu, Xiaoping Tian, Songqi Tang, Yunhui Chen

**Affiliations:** ^1^ School of Pharmacy/School of Acupuncture and Moxibustion/School of Basic Medicine/School of International Education, Chengdu University of Traditional Chinese Medicine, Chengdu, China; ^2^ West China Hospital/West China School of Medicine/State Key Laboratory of Biotherapy, Sichuan University, Chengdu, China; ^3^ Institute of Chinese Medical Sciences, University of Macau, Macau, China; ^4^ School of Chinese Medicine, Hainan Medical University, Macau, China

**Keywords:** flipped classroom, effectiveness, Chinese pharmacy education, meta-analysis, systematic review

## Abstract

**Background:** The application of flipped classroom (FC) pedagogy has recently become increasingly popular in Chinese pharmacy education. However, its effectiveness in improving student learning has not yet been assessed. This study aimed to evaluate the effects of teaching with such pedagogical approach by examining studies that compare the FC approach with the traditional lecture-based learning (LBL) module through a systematic review and meta-analysis.

**Methods:** Seven databases, including the PubMed, EMBASE, Cochrane Library, China National Knowledge Infrastructure, Chinese Scientific Journals Database, Chinese Wanfang database, and China Biomedical Literature Database, were searched from the inception to 30 June 2021, to identify eligible articles of randomized controlled studies. The primary outcomes included the theoretical and experimental test scores, and the secondary outcomes were the results from questionnaires about the number of students who preferred the FC or endorsed its improving effects on their learning enthusiasm, self-learning ability, thinking skills, communication skills, and learning efficiency. The quantitative synthesis was conducted with Revman V.5.3 software following the Cochrane Reviewer’s Handbook guidelines and the Preferred Reporting Items for Systematic Reviews and Meta-Analyses statement.

**Results:** Eleven eligible studies published from 2017 to 2020 enrolling 1,200 students were included in this meta-analysis. The quantitative synthesis demonstrated that the FC module presented an overall more significant effectiveness over traditional LBL approach for Chinese pharmacy education in improving student academic performance as measured by theoretical test scores (SMD = 1.08, 95% CI: 0.60–1.56, *p* < 0.00001) and experimental test scores (MD = 6.62, 95% CI: 4.42–8.82, *p* < 0.00001). Further sub-group analysis revealed that the preferable effectiveness of FC was also evident in both theory-oriented (SMD = 0.77, 95% CI: 0.10–1.45, *p* < 0.00001) and experiments-oriented courses (MD = 6.52, 95% CI: 3.48–9.56, *p* < 0.00001) for both undergraduate (SMD = 0.84, 95% CI: 0.31–1.37, *p* < 0.00001) and 3-year junior-college students (MD = 8.17, 95% CI: 6.44–9.89, *p* < 0.00001). Additionally, analysis on the questionnaire outcomes revealed that more respondents preferred for FC and endorsed its improvement effects on developing students’ learning enthusiasm, self-learning ability, thinking skills, communication skills, and learning efficiency.

**Conclusion:** Current evidence suggests that FC pedagogical approach can effectively improve student learning outcomes and is applicable to Chinese pharmacy education.

## 1 Introduction

Chinese pharmacy education plays a pivotal role in response to the increasing demands for pharmaceutical and medical professionals, and its framework has long been dominated by the traditional lecture-based learning (LBL) pedagogy (Zhou et al., 2016; Lang et al., 2019). A typical LBL classroom is occupied with didactic lectures, and students are expected to listen to the lectures in class and complete their homework after class. With the development of teaching conception, such pedagogy has been considered a teacher-centered passive learning mode, and students are relatively passive during the process of knowledge acquisition (Shi et al., 2015; Faisal et al., 2016; Zeng et al., 2020). In recent years, a growing number of studies have reported the LBL mode is not conducive to helping students improve academic performance, promote learning motivation, develop autonomous learning abilities, and cultivate innovative thinking skills (Nilson, 2016; Fu et al., 2022). Additionally, long-term exposure to simplex and cramming didactic lecturing may contribute to students’ sedentary study styles and introverted and quiet personalities (Lang et al., 2019). Hence, pedagogy reformation and innovation have constantly been proceeding in Chinese pharmacy education during the last decade (Dearnley et al., 2018; Hew & Lo, 2018; Jin & Bridges, 2014).

A flipped classroom (FC, also known as flipped learning or inverted classroom) is a blended learning model that originated from the concepts of constructivism and student-centered learning (Flumerfelt and Green 2013; Smit et al., 2014). In a flipped classroom, the traditional teaching method is carried out by requiring students to obtain background knowledge through viewing lecture materials (e.g., videos, powerpoints, notes, pre-class exercises/quizzes) prior to class, and the conventional in-class didactic teaching is replaced by student-centered interactive activities (Rui et al., 2017; Zhang et al., 2019). Instead of passively sitting and listening in the conventional didactic classroom, students are expected to clarify doubts, ask questions, articulate ideas, and solve problems actively in the precise in-class time to consolidate and enrich their learning and apply what they have learned (Foldnes, 2016). As an innovative and interactive pedagogical approach that incorporates theory, practice, and innovation, the FC pedagogy gives full play to students’ subjective initiatives and has been widely adopted by various disciplines of Chinese pharmacy educators in their curricula for undergraduates and 3-year junior college students (Hurtubise et al., 2014; Li et al., 2016; Persky and McLaughlin., 2017).

In recent years, multiple lines of research have suggested that the FC approach can improve students’ academic performance in various Chinese pharmacy curricula, inspire students to be more enthusiastic learners, empower them to think independently and encourage them to communicate and cooperate skillfully (Hu et al., 2017; Wang et al., 2019, 2020; Kang et al., 2020; Ma et al., 2020). Although the FC approach shows enormous promises for Chinese pharmacy education, its overall effect on improving student learning remains unexamined. Therefore, this meta-analysis was performed to systematically evaluate the effectiveness of FC over the LBL approach in Chinses pharmacy education and hopefully provide some useful information for educators, learners, and investigators concerned.

## 2 Materials and methods

This systematic review and meta-analysis was performed following the guidelines in the Cochrane Handbook for Systematic Reviews of Interventions (Higgins & Green, 2014) and the Preferred Reporting Items for Systematic Reviews and Meta-Analyses Protocols (PRISMA-P) (Moher et al., 2009).

### 2.1 Data sources and search strategies

Two reviewers (WP and YX) independently searched seven electronic databases, including the PubMed, EMBASE, Cochrane Library, China National Knowledge Infrastructure, Chinese VIP information database, Chinese Wanfang Database, and Chinese Biological Medicine Database, from inception to 30 June 2021, without restrictions on language to identify relevant studies. The following terms were used in a combination for the electronic search: flipped classroom, flipped class, flipping the classroom, flipped learning, flipped instruction, inverted classroom, FC, Chinese Materia Medica, Chinese pharmacy, pharmaceutical, Chinese Medicine, traditional Chinese medicine, herbal medicine, comparative study, comparison, randomized control, and randomization. The search strategy for PubMed is presented in Table 1, and corresponding modifications were made to accommodate the requirements of other databases. In addition, manual searches were performed to the references of retrieved studies. Any inconsistency was resolved by consulting the third reviewer (YHC).

**TABLE 1:** Search strategy for the PubMed.

No.	Search terms
#1	Flipped classroom or flipped class or flipping the classroom or flipped learning or flipped instruction or inverted classroom or FC
#2	Medicine, Chinese traditional (mesh terms)
#3	Pharmaceutical preparations (mesh terms)
#4	Herbal medicine (mesh terms)
#5	Chinese material medical or Chinese pharmacy or pharmaceutical or Chinese medicine or traditional Chinese medicine or herbal medicine
#6	#2 or #3 or #4 or #5
#7	Randomized controlled trial [pt]
#8	Randomly [tiab]
#9	Randomized [tiab]
#10	Comparative study [tiab]
#11	Comparison [tiab]
#12	Trial [tiab]
#13	Groups [tiab]
#14	#7 or #8 or #9 or #10 or #11 or #12 or #13
#15	#1, #6 and #14

### 2.2 Eligibility criteria

The retrieved research was considered eligible when it fulfilled the predefined inclusion criteria as follows: 1) Type of study: randomized controlled trials; 2) Population: students receiving Chinese pharmacy education, regardless of age, gender, ethnicity, nationality, discipline, and major; 3) Intervention: using FC pedagogical approach, either alone or combining with other methods, with a clear description of pre-class and in-class activities; 4) Comparator: using traditional LBL in Chinese pharmacy curricula teaching; 5) Outcome measurements: primary outcomes were the theoretical test scores and the experimental test score; and secondary outcomes included the incidence of students who endorsed the effectiveness of the flipped classroom on improving their comprehensive competency (e.g., learning enthusiasm, self-learning ability, thinking skills, communication skills, and learning efficiency) from the questionnaires. Non-RCTs, non-empirical studies, literature reviews, duplicated publications, subjects other than Chinese pharmacy education, and reports with incomplete or missing datasets or results to calculate effect sizes were excluded.

### 2.3 Study selection and data extraction

Two reviewers (YX and YHC) independently screened the titles and abstracts of the retrieved studies and then reviewed the full text using the pre-specified eligibility criteria. The following information was extracted: study ID, first author, publication year, sample size, the subject curriculum of Chinese pharmacy education, characteristics of the students, information on the FC implementation and the traditional LBL control, and outcome measurements. Any discrepancy was solved by consulting a third reviewer (XPT). All data were cross-checked prior to entry into and analysis with RevMan V.5.3 software (The Cochrane Collaboration, NCC, CPH, Denmark).

### 2.4 Risk of bias assessment

Two reviewers (YX and JWW) independently used the Cochrane risk-of-bias tool for randomized trials to grade the risks of bias as high, unclear, or low risk of bias in terms of the following seven domains: randomization sequence generation, randomization allocation concealment, blinding of participants, blinding of personnel, blinding of outcome assessors, incomplete outcome data, selective reporting, and other bias. A third reviewer (SQT) was consulted for any inconsistency.

### 2.5 Statistical analysis

The RevMan 5.3 software was employed for the quantitative synthesis. A standard mean differences (SMD) or mean differences (MD) with 95% confidence intervals (CIs) was applied for continuous variables, while a risk ratio (RR) with 95% CIs was utilized for dichotomous data. The chi-square statistic and *I* ^2^ statistic were employed to assess statistical heterogeneity. The fixed-effects model was used for a low heterogeneity (*I* ^2^ < 50%), and the random-effects model was applied if heterogeneity was substantial in the pooled studies (*I* ^2^ > 50%). Subgroup analyses were performed to identify the potential source of high heterogeneity and assess the pedagogical effect in two major moderators (e.g., types of students and curriculum). *p* < 0.05 was deemed statistically significant. The publication bias was assessed with a funnel plot when more than ten studies were enrolled. Sensitivity analysis was conducted to evaluate the robustness of the pooled effects by omitting individual studies sequentially.

## 3 Results

### 3.1 Eligible studies

**FIGURE 1:**
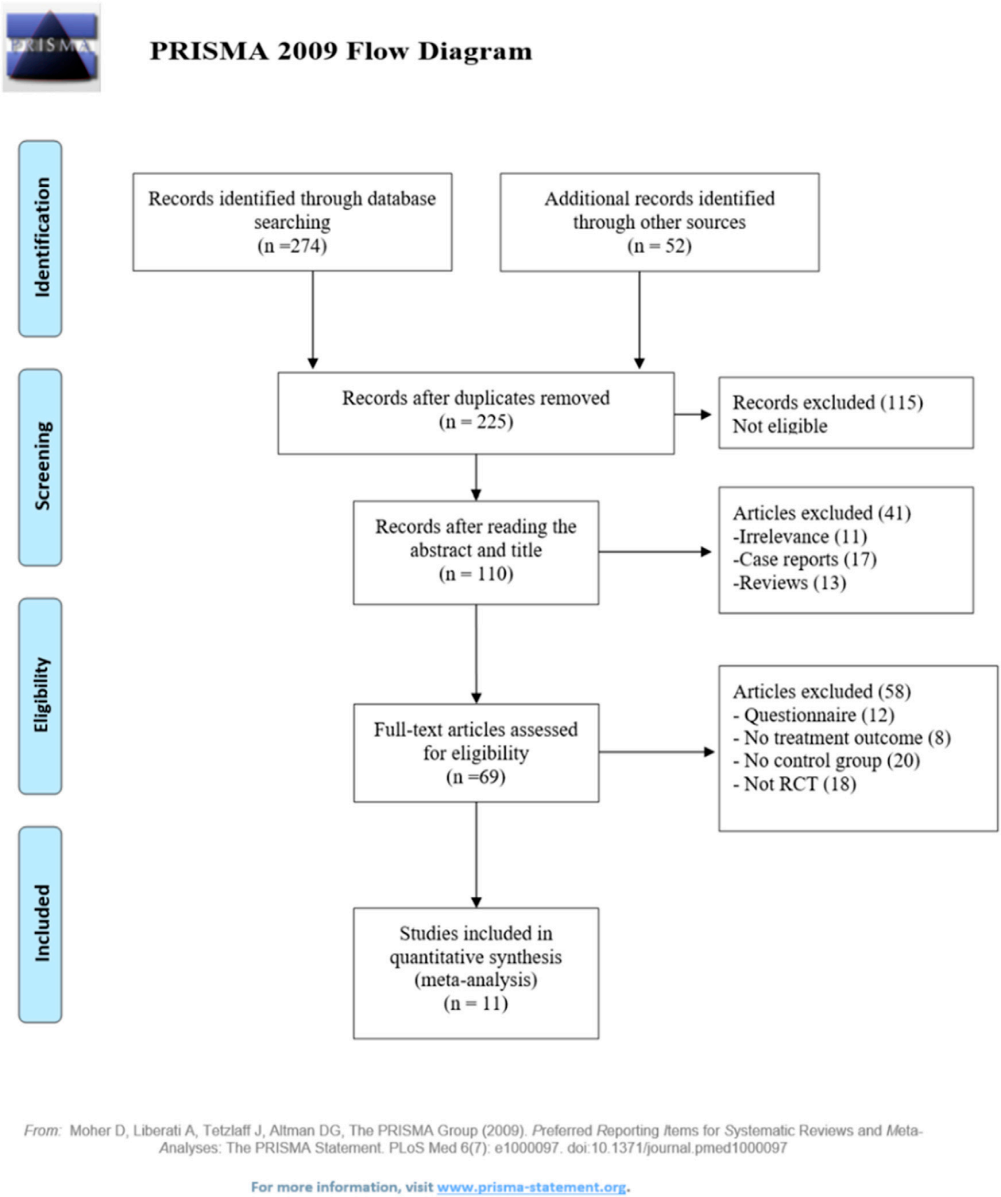
PRISMA flowchart of study selection and identification process.

Initially, 326 studies were identified following the predefined search strategy, and 225 studies remained after duplication deletion. Upon the preliminary review, 115 articles unrelated to Chinese pharmacy education or non-empirical were eliminated. Furthermore, by reviewing the title and abstract of the remains, 41 articles were removed, including 11 irrelevant studies, 17 case reports, and 13 reviews. After the full-text screening, 58 articles were removed, including 12 questionnaires, eight with insufficient data on outcomes or descriptions of the flipping process, 20 without a control group, and 18 non-randomized controlled studies. Eventually, 11 studies were included in this meta-analysis (Hu et al., 2017; Ge et al., 2019; Wang et al., 2019; Dong et al., 2020; Kang et al., 2020; Li et al., 2020; Liu et al., 2020; Ma et al., 2020; Wang et al., 2020; Yang, 2020; Zhu et al., 2020). The flowchart for the selection process of eligible literature is shown in Figure 1.

### 3.2 Characteristics of included studies

Eleven randomized controlled studies enrolling 1,200 participants (601 in the FC pedagogy group and 599 in the traditional LBL control group) were included in this meta-analysis. All the studies were conducted in China and published from 2017 to 2020, ten in Chinese (Hu et al., 2017; Ge et al., 2019; Wang et al., 2019; Yang, 2020; Dong et al., 2020; Kang et al., 2020; Li et al., 2020; Liu et al., 2020; Ma et al., 2020; Zhu et al., 2020) and one in English (Wang et al., 2020).

The FC pedagogical approach was clearly identified in all studies, including eight studies for undergraduate education (4-year program for the pharmaceutical major and 5-year for the medical major) (Ge et al., 2019; Dong et al., 2020; Kang et al., 2020; Li et al., 2020; Liu et al., 2020; Ma et al., 2020; Wang et al., 2020; Zhu et al., 2020) and two for 3-year junior-college education (Hu et al., 2017; Wang et al., 2019). The LBL pedagogy was applied in the control group in all studies. The flipped classroom was adopted in a wide variety of Chinese pharmacy curricula, including theory-oriented curricula in five studies (Kang et al., 2020; Li et al., 2020; Liu et al., 2020; Ma et al., 2020; Wang et al., 2020) and experiment-oriented curricula were clearly stated in four studies (Ge et al., 2019; Wang et al., 2019; Dong et al., 2020; Zhu et al., 2020).

For the outcome variables, ten studies reported the theoretical test score (Hu et al., 2017; Wang et al., 2019; Dong et al., 2020; Kang et al., 2020; Li et al., 2020; Liu et al., 2020; Ma et al., 2020; Wang et al., 2020; Yang, 2020; Zhu et al., 2020) and six studies reported the experimental test score (Hu et al., 2017; Ge et al., 2019; Wang et al., 2019; Yang, 2020; Dong et al., 2020; Zhu et al., 2020). In addition, questionnaires were employed in two studies to assess students’ preference for the FC (Wang et al., 2019; Kang et al., 2020), in five studies for evaluating the effects of such pedagogies on improving students’ learning enthusiasm (Dong et al., 2020; Ge et al., 2019; Hu et al., 2017; Wang et al., 2020; Yang, 2020), four studies for self-learning ability (Dong et al., 2020; Hu et al., 2017; Wang et al., 2020; Yang, 2020), two studies for thinking and communication skills (Hu et al., 2017; Dong et al., 2020), one study for cooperative ability (Hu et al., 2017), and one study for learning efficiency (Yang, 2020). The characteristics of the included studies are summarized and presented in Table 2.

**TABLE 2:** Characteristics of the included studies.

Study	Curriculum	Student major/Degree	Student equivalence	Instructor equivalence	Sample size (interv./Cont.)	Interv	Cont	Outcome measurements
Wang et al. (2019)	Pharmaceutics of Chinese	Science of Chinese Pharmacy/Junior college student	NSSD	NR	20/20	FC (availability of pre-class video/reading/learning assignment + in-class assignment-based discussion/student presentation/instructor feedback/experiment)	LBL	①+②+③
	Pharmacy- Experiment							
Kang et al. (2020)	Science of Chinese Pharmacy	Traditional Chinese Medicine/Undergraduate	NSSD	NR	67/67	FC (pre-class video/reading/learning assignment/exercise + in-class problem-based lecturing/discussion/quiz)	LBL	①+③
Ma et al. (2020)	Chemistry of Chinese Pharmacy	Science of Chinese Pharmacy/Undergraduate	NR	NR	34/28	FC (pre-class video/reading/exercise/learning assignment + in-class lecturing/student presentation/teacher feedback/Q&A)	LBL	①
Hu et al. (2017)	Applied Chinese Pharmacy	Science of Pharmacy/Junior college student	NSSD	NR	53/54	FC (pre-class video/reading/exercise+ in-class Q&A/student presentation/teacher- and student-student comments)	LBL	①+②+④
Wang et al. (2020)	Medical Statistics	Medicine/Undergraduate	NSSD	Identical	44/44	FC (pre-class video/reading/exercise or quiz + in-class case discussion/learning assignment/Q&A/data analysis project)	LBL	①+④
Liu et al., 2020 ^a^	Formulae of Chinese Medicine	Science of Chinese Pharmacy/Undergraduate	NSSD	Identical	39/46	FC (pre-class video/reading/exercise+ in-class student lecturing/teacher commenting/Q&A)	LBL	①
Liu et al., 2020 ^b^	Formulae of Chinese Medicine	Integrated Traditional Chinese and Western Medicine/Undergraduate	NSSD	Identical	50/50	FC (pre-class video/reading/exercise+ in-class student lecturing/teacher commenting/Q&A)	LBL	①
Zhu et al. (2020)	Chemistry of Chinese Pharmacy—Experiment	Science of Chinese Pharmacy/Undergraduate	NSSD	Identical	57/54	FC (pre-class video/reading + in-class presentation/assimilation/discussion/Q&A/experiment)	LBL	①+②
Li et al. (2020)	Pharmaceutical Botany	Science of Chinese Pharmacy/Undergraduate	NR	Identical	51/50	FC (pre-class reading/learning assignment + in-class teacher lecturing/Q&A/discussion)	LBL	①
Yang, (2020)	Drug Quality Inspection Technology	Pharmacy Science/NR	NSSD	NR	100/100	FC (pre-class video-massive online open courses/reading/exercise + in-class discussion/practice)	LBL	①+②+④
Dong et al. (2020)	Pharmacology—Experiment	Clinical Medicine/Undergraduate	NSSD	NR	51/52	FC (pre-class video/reading + in-class discussion, Q&A/experiment/teacher feedback)	LBL	①+②+④
Ge et al. (2019)	Pharmacology of Chinese Pharmacy—Experiment	Science of Chinese Pharmacy/Undergraduate	NR	Identical	35/34	FC (availability of pre-class video/reading/learning assignment/Q&A+ in-class problem-based discussion/experiment)	LBL	②+④

NSSD, no statistically significant difference in baseline data; IIs, identical instructors; Interv, intervention; Cont, control; FC, flipped classroom; PBL, problem based learning; LBL, lecture-based learning; TBL, team-based learning; TCM, traditional Chinese medicine; PAD, Presentation-assimilation-discussion; NR, not reported; ①: theoretical test score; ②: experimental test score; ③student preference for FC, over LBL; ④ comprehensive abilities including learning motivation, self-learning, thinking, and communication.

a One study with two independent datasets for different majors Liu et al., 2020.

b One study with two independent datasets for different majors Liu et al., 2020.

### 3.3 Risk of bias assessment

**FIGURE 2:**
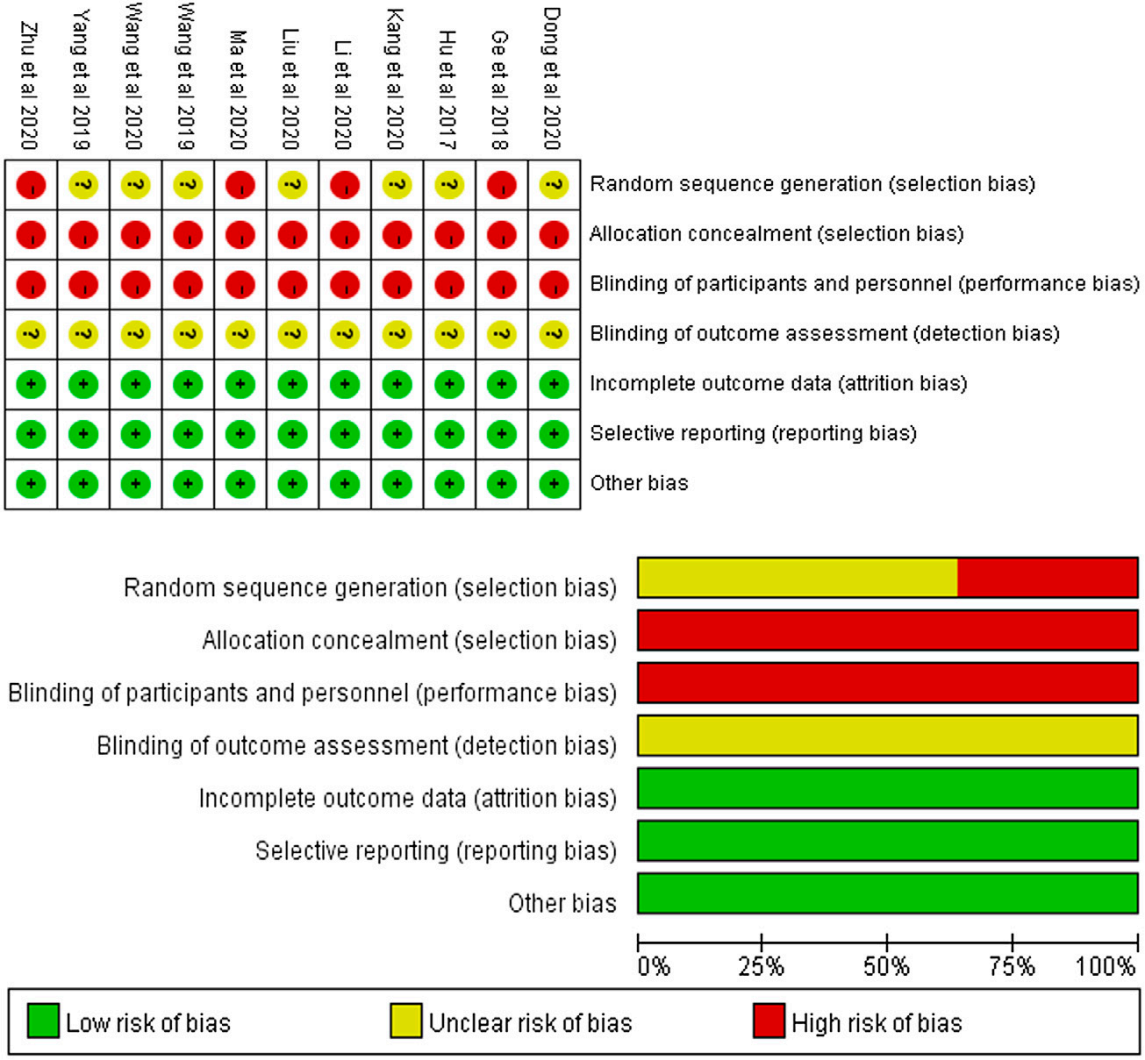
Assessment of methodological quality by the Cochrane risk-of-bias tool.

Using the Cochrane risk-of-bias tool for randomized trials, seven studies mentioned randomization but did not describe the generation method (Hu et al., 2017; Wang et al., 2019; Yang, 2020; Dong et al., 2020; Kang et al., 2020; Liu et al., 2020; Wang et al., 2020), and four studies did not mention randomization and were rated as high risk (Ge et al., 2019; Li et al., 2020; Ma et al., 2020; Zhu et al., 2020). None of the studies reported the allocation concealment procedure. Given the characteristics of such pedagogy methods, the participants and personnel could not be blinded in any of these studies. All the studies had complete data, and hence the attrition bias was assessed as low risk. The risk of bias assessment is summarized and shown in Figure 2.

### 3.4 Effect of flipped classroom pedagogy on improving student learning outcomes

#### 3.4.1 Theoretical test scores

**FIGURE 3:**
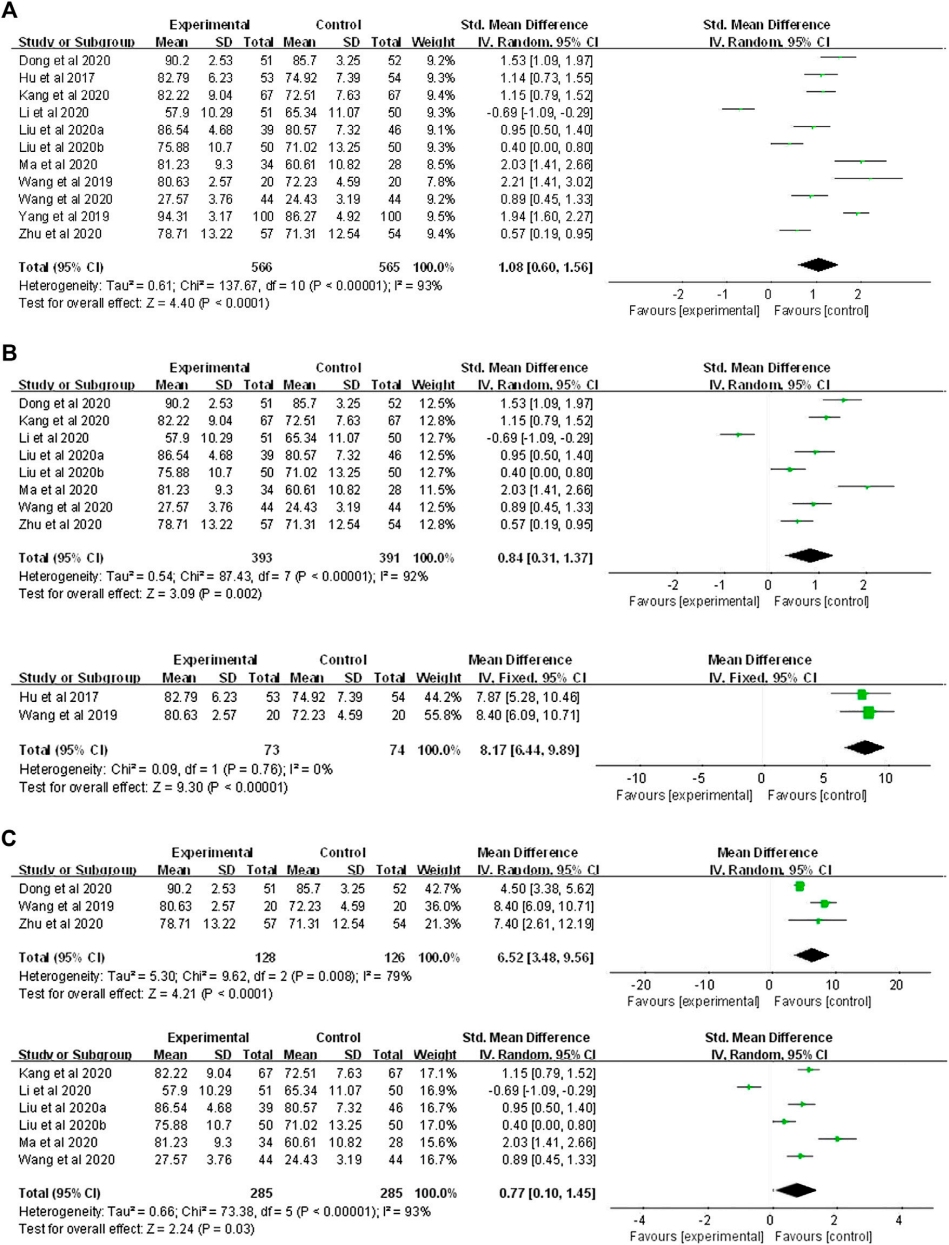
Forest plot for effectiveness of FC versus LBL in **(A)** theoretical test score; **(B)** undergraduate versus 3-year junior-college student subgroup analysis **(C)** theory- and experimental-oriented curriculum subgroup analysis.

Ten of eleven studies involving 1,131 participants (566 in the FC group and 565 in the LBL group) reported the theoretical test score. The pooled data of the meta-analysis using a random-effects model showed an overall significant effect in favor of the FC approach for Chinese pharmacy curricula as measured by increased theoretical test scores (SMD = 1.08, 95% CI: 0.60–1.56, *p* < 0.00001) (Figure 3A).

Subgroup analyses were performed based on different types of students and curricula. Participants were clearly reported to be undergraduates in seven studies (393 in the FC group and 391 in the LBL group) and 3-year junior-college students in two studies (73 in the FC group and 74 in the LBL group). The aggravated results of meta-analysis using the random-effects model revealed that the FC was beneficial to improve the academic performance of both undergraduate (SMD = 0.84, 95% CI: 0.31–1.37, *p* < 0.00001) and junior-college students (MD = 8.17, 95%CI: 6.44–9.89, *p* < 0.00001) theoretically (Figure 3B).

Further, subgroup analysis was carried out for different types of curricula, as the course was clearly stated to be experiment-oriented in three studies (128 in the FC group and 126 in the LBL group) and theoretical-oriented in five studies (285 in the FC group and 285 in the LBL group). The pooled results of meta-analysis applying the random-effects model demonstrated that compared with the LBL groups, the FC could significantly improve student knowledge gain for both experiment-oriented (MD = 6.52, 95%CI: 3.48–9.56, *p* < 0.00001) and theoretical-oriented (SMD = 0.77, 95% CI: 0.10–1.45, *p* < 0.00001) courses of Chinese pharmacy curricula (Figure 3C).

#### 3.4.2 Experimental test scores

**FIGURE 4:**
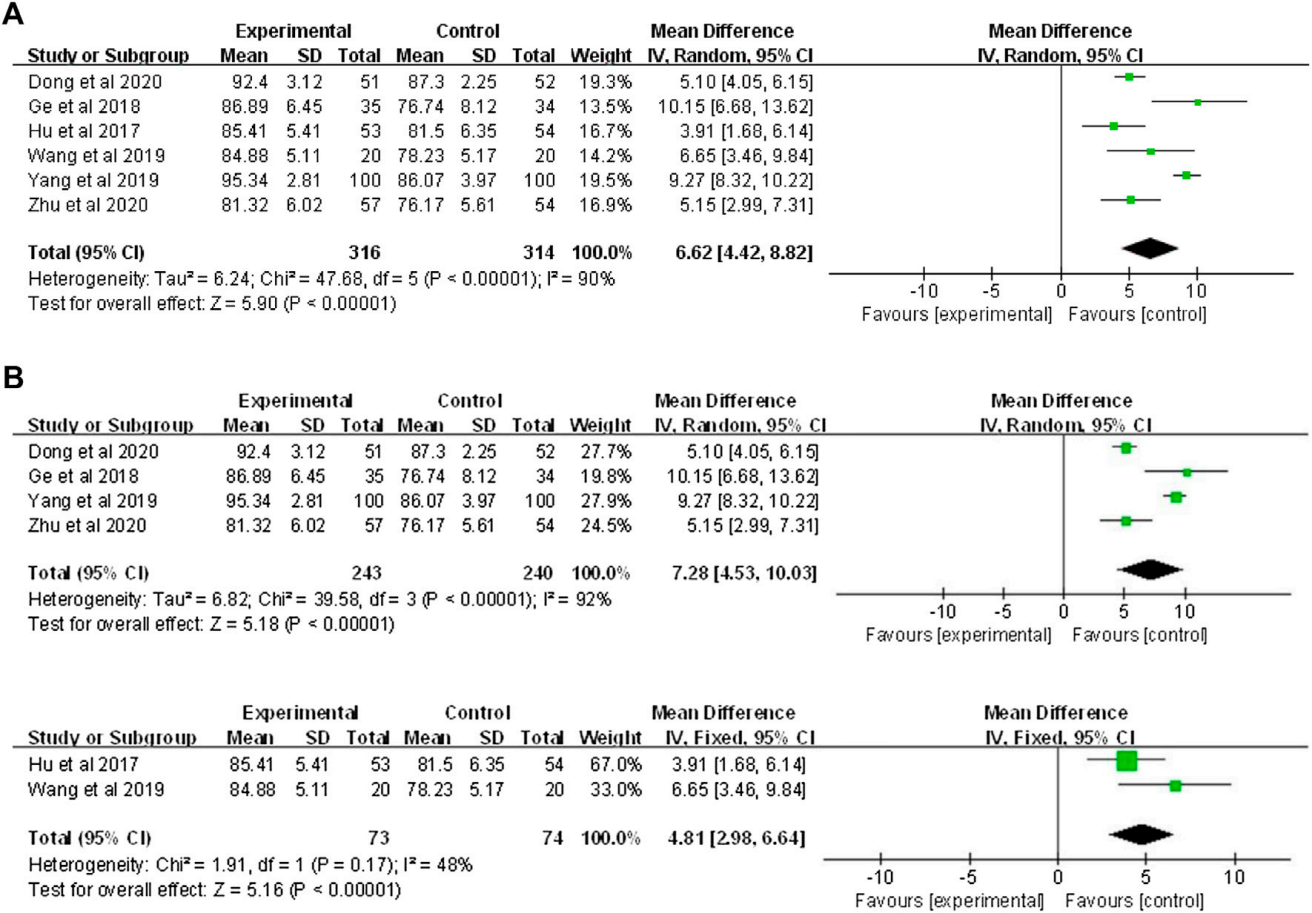
Forest plot for effectiveness of FC versus LBL in **(A)** experimental test score; **(B)** undergraduate versus 3-year junior-college student subgroup analysis.

Six studies involving 630 participants (316 in the FC group and 314 in the LBL group) reported the experimental test scores. The pooled data of the meta-analysis using the random-effects model showed that the FC significantly improved the experimental test scores when compared with the LBL (MD = 6.62, 95%CI: 4.42–8.82, *p* < 0.00001) (Figure 4A). Further subgroup analysis also demonstrated that the favorable effectiveness of FC on enhancing experimental capability for both undergraduates (MD = 7.28, 95% CI: 4.53–10.03, *p* < 0.00001) and 3-year junior-college students (MD = 4.81, 95% CI: 2.98–6.64, *p* < 0.00001) (Figure 4B).

#### 3.4.3 Comprehensive competency

**FIGURE 5:**
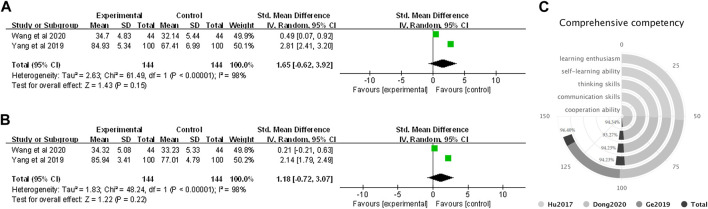
Forest plot for effectiveness of FC versus LBL in improving students’ comprehensive competency of **(A)** learning motivation; **(B)** self-study ability; and narrative analysis of **(C)** learning enthusiasm, self-learning ability, thinking and communication skills, and cooperation ability.

Two studies involving 288 students (144 in FC and 144 in LBL) utilized scored surveys to explicitly compare the FC and LBL pedagogies in improving student enthusiasm and self-learning ability. The pooled data from questionnaires indicated that the introduction of FC pedagogy developed more students’ learning enthusiasm (SMD = 1.65, 95% CI: −0.62 to 3.92, *p* = 0.15) (Figure 5A) and self-study ability (SMD = 1.18, 95% CI: −0.72 to 3.07, *p* = 0.22) (Figure 5B), however, the statistical significance was not significant. In addition, three studies applied survey questionnaires to assess student acknowledgment of the FC in improving comprehensive competency. Narratively, overall endorsement for FC pedagogy in improving learning enthusiasm was reported by 134/139 respondents (96.40%), self-learning ability by 98/104 respondents (94.23%), thinking skills by 98/104 respondents (94.23%), communication skills by 97/104 respondents (93.27%), and cooperation ability by 50/53 respondents (94.34%) (Figure 5C).

### 3.5 Publication bias assessment

**FIGURE 6:**
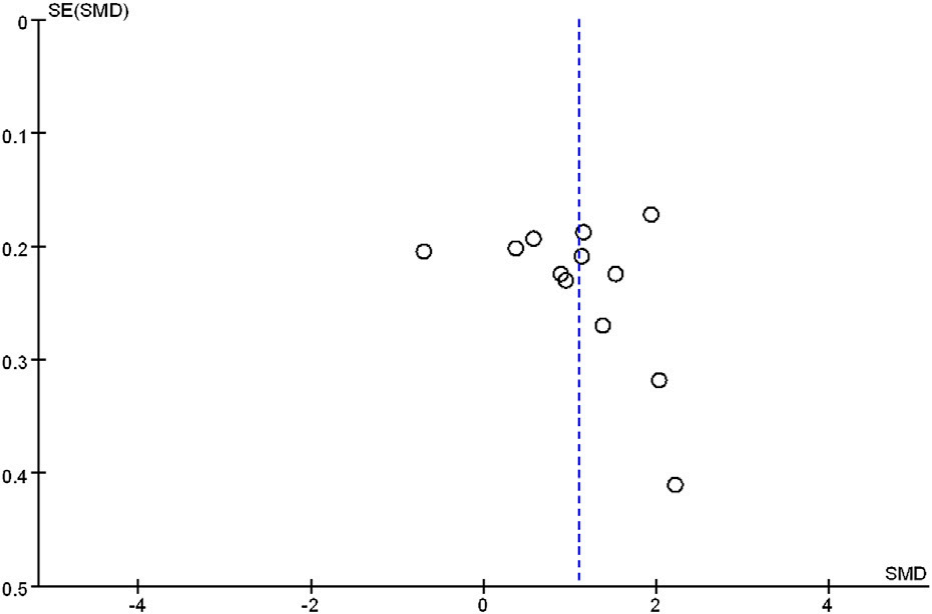
Funnel plot of FC versus LBL in theoretical test scores (Egger’s test, *p* > 0.05)

The funnel plot was utilized to assess the publication bias. The results showed that the theoretical test score was slightly asymmetrical, combined with the value of Egger’s test (*p* > 0.05), indicating the possibility of publication bias was minor (Figure 6).

### 3.6 Sensitivity analysis

**FIGURE 7:**
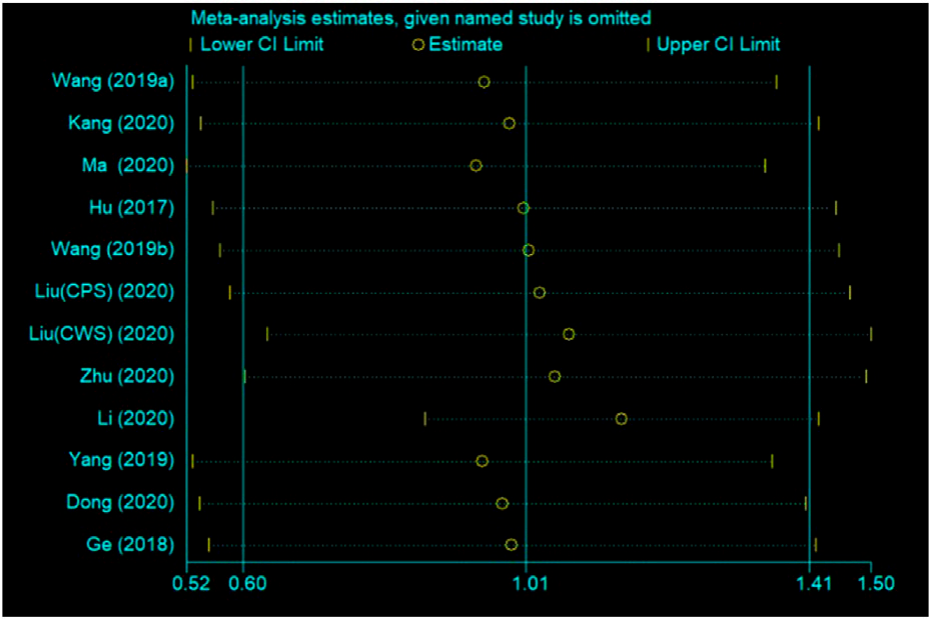
Sensitivity analysis of FC versus LBL in theoretical test scores.

The sensitivity analysis was carried out to the theoretical test score by sequentially eliminating individual studies. The results showed that the pooled effect remained unchanged, indicating the stableness and robustness of the pooled results (Figure 7).

## 4 Discussion

Chinese pharmacy education intends to equip students with the knowledge and skills necessary to become qualified professionals and also help them develop comprehensive competence to flourish in their future careers (Kuang, 2015; Peng 2021). As an educational innovation based on student-centered learning theories and active learning strategies, the FC pedagogical approach has been wildly used in Chinese pharmacy education, covering various disciplines at different hierarchical levels of higher education. Although several systematic reviews have been performed to assess the effect of FC application on various courses, such as clinical medicine (traditional Chinese medicine and western medicine), radiology, pharmacology, and nursing (Hew and Lo., 2018; Ge et al., 2020; Li et al., 2020; Wang et al., 2020; Chen et al., 2022), the present study, to the best of our knowledge, was the first meta-analysis to evaluate the evidence concerning the overall effectiveness of the FC pedagogical approach in improving student learning in Chinese pharmacy education compared with traditional LBL using multiple academic online databases.

In this meta-analysis, 11 comparative studies published from 2017 to 2020 covering nine curricula of Chinese pharmacy education were included. The quantitative synthesis demonstrated that the FC module, either alone or with other teaching approaches, presented more significant effectiveness over the traditional LBL approach in improving student academic performance as measured by theoretical and experimental test scores. Subgroup analyses were further performed to explore the effectiveness of such pedagogical methods in different categories of students and types of curricula. Encouragingly, the results indicated that both undergraduates and 3-year junior-college students experienced considerable improvements after being taught with FC. Its preferable effectiveness was also evident in both theory-oriented and experiments-oriented Chinese pharmacy curricula. In addition, studies that applied survey questionnaires to assess student endorsement of the FC module in improving their comprehensive competence were analyzed. The results suggested that FC pedagogy might enhance students’ learning enthusiasm, self-learning ability, thinking skills, communication skills, and cooperative ability.

FC effectiveness in Chinese pharmacy education may attribute to multiple factors. First, it provides students unlimited access to the pre-recorded video lectures before class and makes personalized learning possible with more flexibility. Students can learn anywhere at their own pace (Lin et al., 2017; Hew & Lo, 2018; Wang et al., 2020; Zheng and Guo, 2020) and may watch the videos multiple times to thoroughly understand a particular subject when necessary. Second, it alters the sedentary in-class dynamics of focusing on how much knowledge can be absorbed by passively listening and requires students to apply their knowledge in class, allowing them to become more actively engaged with the course (Dearnley et al., 2018; Cotta et al., 2016; Lin et al., 2017). Third, flipping the classroom increases both teacher-student and student-student interaction, and students are encouraged to interact and cooperate with their peers (Wei 2021). It offers peer-to-peer grouping study opportunities that may enhance the mastery of relative knowledge and comprehensive competency. Last but not least, students are presumably experienced more attention lapses in traditional lecturing classrooms (Bradbury 2016), while flipping the classroom can engage students for a longer period and thus may improve learning outcomes.

Although the effectiveness of the FC pedagogical approach in Chinese pharmacy education was meta-analyzed, this study has several limitations: 1) given the characteristics of teaching process, allocation concealment and participants blinding were unrealizable, which may carry a substantial risk of overrating the effectiveness of such pedagogy; 2) considerable heterogeneity was notified in the pooled results of theoretical and experimental test scores, and the potential reasons might be different baseline conditions in each included study and diverse test-design frameworks across various Chinese pharmacy curricula; 3) although five of the studies included clearly stated that the “identical” teachers provided the FC and LBL instruction, the difference in teachers’ levels across all the studies might also contribute to the heterogeneity; 4) no unified and standardized questionnaires were utilized to evaluate the effectiveness of such pedagogy in improving learning enthusiasm, self-learning ability, thinking and communication skills, and cooperative ability, which may underestimate FC’s value in enhancing students’ comprehensive competency; and 5) the literature was searched without language restriction; however, all the publications were from China, and the funnel plot indicated the minor existence of publication bias.

This study revealed that FC could significantly improve students’ academic performance and comprehensive ability compared with the traditional LBL teaching method. It may provide a valuable evidence-based basis for the ongoing reform of higher education. To better utilize the results of this meta-analysis in a real teaching setting, instructors need to be trained systematically in advance as FC pedagogy may put forward higher requirements for teachers’ ability, requiring them to search for high-quality course resources before class, while also designing in-class Q&A programs and teaching activities according to students’ pre-class learning and related thinking. In addition, well-designed and high-quality studies may be warranted to tackle some issues unresolved by current studies, including: 1) how much pre-class workload is optimal for learning outcome improvement; 2) whether the style of pre-recorded video influences learning outcomes; 3) whether the FC approach can exert positive longitudinal effects on student professional careers by follow-up studies.

## 5 Conclusion

In summary, this study demonstrates that the FC pedagogical approach can significantly improve students’ academic performance and comprehensive competencies compared with traditional LBL methods. It might be considered a promising teaching strategy for conducting Chinese pharmacy education.

## Data Availability

The original contributions presented in the study are included in the article/Supplementary Material, further inquiries can be directed to the corresponding authors.
